# 1605. Dual Therapy VS Triple Therapy in Experienced PLWH: a Multicentre One-Year Long Observational Study

**DOI:** 10.1093/ofid/ofad500.1440

**Published:** 2023-11-27

**Authors:** Ylenia Russotto, Antonino Gaspare Saia, Emmanuele Venanzi Rullo, Giovanni Francesco Pellicanò, Giuseppe Nunnari

**Affiliations:** Unit of Infectious Diseases, Department of Clinical and Experimental Medicine, University of Messina, 98124 Messina, Italy, Messina, Sicilia, Italy; Unit of Infectious Diseases, Department of Clinical and Experimental Medicine, University of Messina, 98124 Messina, Italy, Messina, Sicilia, Italy; Unit of Infectious Diseases, Department of Clinical and Experimental Medicine, University of Messina, 98124 Messina, Italy, Messina, Sicilia, Italy; Unit of Infectious Diseases, Department of Adult and Childhood Human Pathology “Gaetano Barresi”, University of Messina, 98124 Messina, Italy, Messina, Sicilia, Italy; Unit of Infectious Diseases, Department of Clinical and Experimental Medicine, University of Catania, 95131 Catania, Italy, Catania, Sicilia, Italy

## Abstract

**Background:**

The introduction of new antiretroviral drugs with increased viral potency, as integrase inhibitors (INI), has changed the history of HIV. The higher potency has brought to light the necessity to reduce drug adverse effects, drug-drug interaction and costs. We conducted a multicentre observational study and we observed a group of people living with HIV (PLWH) who had switched to a two drugs regimen (2DR) for a year, comparing it with a group of PLWH who maintained the 3DR regimen, focusing on inflammation markers.

**Methods:**

We did a multicentre parallel-group, observational study, from four Sicilian hospitals: “Gaetano Martino” hospital of Messina, “Cutroni-Zodda” hospital of Barcellona Pozzo di Gotto, “Papardo” hospital of Messina, “Cannizzaro” hospital of Catania. First group comprehends PLWH aged 18 years or older, who switched from a 3 or more drugs antiretroviral therapy (ART), of any class, to a 2DR ART, of any class excluded long acting treatment with cabotegravir + rilpivirine, between 2020 and 2022. Second group comprehends PLWH aged 18 or older, who maintained their 3DR, of any class. We performed blood analyses at three time-points (time 0, 6 months and 12 months).We considered the following items: HIV viral load, CD4+, CD4+/CD8+, platelets (PLT) count, C reactive protein (CRP) and lactate dehydrogenase (LDH). We performed a statistical analysis using the student T test.

**Results:**

Group one comprehends 81 individuals, 69 males, mean age of 54 ± 8 years old. Group two comprehends 97 individuals, 55 males and 43 females, mean age of 49 ± 11 years old. No statistically significant difference between 2DR group and 3DR group was found in terms of HIV viral load (p-value 0.203), CD4+ count (p-value 0.238), CD4+/CD8+ (p-value 0.556), PLT count (p-value 0.631), LDH (p-value 0.295) and CRP (p-value 0.558). No adverse effects to the therapy were reported.

**Conclusion:**

The major concern in switching to 2DR is the diminished pressure on HIV and increase of inflammation factors. Recent studies have reported no changes in inflammation markers in HIV-suppressed people who switch to 2DR, compared to those who maintain the 3DR. Considering the brief time of observation, we did not observe statistically significant changes in inflammation markers nor viral load, similarly to other studies.

**Disclosures:**

**All Authors**: No reported disclosures

